# From Land to Sea; Embracing a Renewable Future

**DOI:** 10.4172/2155-952X.S6-003

**Published:** 2014-04-05

**Authors:** Harry T Whelan, Heather Annis, Phillip Guajardo

**Affiliations:** Medical College of Wisconsin 8701 W, Watertown Plank Rd, CCC 540 Milwaukee, WI 53226, USA

**Keywords:** Bioresources, Energy, Pharmaceuticals, Nutrition, Marine biodiversity

## Abstract

The authors discuss the ever increasing role of biological renewable resources in energy, nutrition, and pharmaceuticals; specifically those potentially available deep within the oceans. They provide a list of products already gleaned from this vastly untapped marine environment; discuss the innovations in technology required to effectively explore and prospect the deeper reaches of the ocean; expose the impressive contribution to the economy; and expound the paramount importance of protecting the oceans to ensure the future. Already many new proteins, enzymes, and pharmaceuticals are being developed from the fauna and flora of the forests and relatively shallow economic zones of the ocean. With much of the ocean still an unexplored frontier, the authors hope to promote increased interest in research and development in this arena.

## Dawn of Biology

As the issues of global warming and use of fossil fuels become more important to consumers around the globe, countries are rising to meet the challenge of embracing renewable resources. In Sweden, for example, they propose having a renewable resource dependent road system by 2025 [1] or China, which in 2005 passed the Renewable Energy Law, making it the largest government sponsored commitment to renewable energy in the world [2]. Researchers from energy, to plastics to pharmaceuticals are finding ways to replace petroleum byproducts with biological alternatives. Over 4000 natural biological organohalogens have been discovered. These compounds are found in products ranging from hormones, and natural pesticides, to antibiotics [3]. Whilst there are regularly new discoveries in flora and fauna from the Amazon to Vietnam, the deep ocean remains largely untapped so it is logical to presume that great advances in pharmaceuticals, energy, and consumables loom on the horizon as we transition from land to sea.

## Wealth of the Sea

The ocean comprises 78.5–97% of Earth’s biosphere with only a tiny fraction explored [4]. One particular area of interest is the Marianas Trench in the Pacific Ocean, seven miles deep and easily able to fit Mt. Everest with over a mile to spare. The variety of ocean habitats means that organisms have adapted to a multitude of unique environments. As such, the abundance of novel and adapted organisms should yield many new genes that hold the key to their adaptive abilities. Adaptation to a specific ocean environment can come in many forms. For example, fat accumulation that typically aids in buoyancy is seen in organisms ranging in size from microscopic to quite large. Plankton has a large surface area and appendages that increase drag, aiding in buoyancy, while fish use air bladders [5]. In hospitable hot water vents, the crushing depths of the Marianas trench, and the pitch darkness of the ocean floor—all hold living creatures whose genes code for adaptive proteins. Harvesting these organisms, and obtaining their genes, holds the key to commercially significant biomaterials.

The sea has proven an inhospitable environment for humans. Denizens of the deep have specific adaptations that allow them to live in this challenging area. Pinnipeds and otters have the ability to super concentrate NaCl in urine allowing them to drink from the ocean with no net loss of free water [6]. Investigation of marine animal’s fluid and electrolyte homeostasis may open the possibility of safe and physiologic human consumption of seawater. This technology could lead to a drastic change in our ability to live in and on the ocean in the future.

Organisms from cold climes often have specific genetically encoded adaptations that confer a survival in frigid temperatures. Minor alterations in amino acid sequence results in a crucial differences. Cold can adversely affects nerve conduction but Antarctic fish continue to conduct at –50°C. At these temperatures one might expect that fish would freeze however this is countered by the production of special peptides and glycopeptides that act as a natural form of antifreeze. Following hepatic synthesis, molecules of this biological antifreeze enter the blood and extracellular fluid where they prevent ice formation. Commercial possibilities for this natural antifreeze are countless [7].

Some useful microorganisms extracted from the ocean include a Vibrio species that produces an exopolysaccharide that is similar to heparin and a Pseudomonas species that removes cadmium from solution by precipitating it on the cell wall. The latter has promise for heavy metal removal from the environment during toxic and hazardous spills [8].

Currently, there are few medicines that have been developed and placed on the market from sea organisms. However, the pipeline to these potential interventions is expanding at a rapid rate with thousands of drugs being developed from a variety of organisms like mollusks, snails and fish [9].

Antimicrobials are well represented among marine natural products. The ocean floor is home to a group of actinomycetes, bacteria that in their terrestrial form have been used in the development of many different antibiotics. (UCSD Science Daily 2003) A new potential antibiotic, Salinosporamide A, has been found that may have the ability to inhibit tumor growth such as breast, colon, and non-small cell lung cancer [10]. Larger organisms such as sponges of the Tethya and Halichondria families have been utilized in the production of chemotherapy agents, antibiotics and antivirals [11].

Fish, too, have added a microscopic contribution. One novel class of chemicals found in fish is the CAP or Cationic Antimicrobial Peptide. These are proteins that help defend the fish from microbial invaders. CAP’s have a complex mechanism of action that includes destruction of microorganism as well as augmentation of host immune function. CAP’s have been shown to have broad antibacterial activity, as well as some propensity for activity against viruses, fungi, protozoa, and malignant cells. Other promising aspects of CAP’s include their binding of exotoxin with a concomitant decrease in the likelihood of sepsis [10].

Another major contributor from the oceans is invertebrates supplying ‘new marine natural products from invertebrates’. (NMNPI) More than 10,000 NMNPI have been discovered since 1990 [12]. The development of antifungal and anti-tuberculosis agents can give hope to immunocompromised patients. Jasplakinolide, also called Jaspamide, is derived from a Fijian sponge and used in mice with vaginal yeast infection, it had comparable actions to miconazole [13]. Another antifungal, Ircinol A is especially promising because of its low cytotoxicity and potential for side effects [14].

There have been many different medications synthesized from natural products that have anti-cancer properties (i.e. taxoids, vinca alkaloids and anthracyclines). *Trididemnum solidum*, a Caribbean Sea Squirt has been the source of several antitumor agents. Didemnim B inhibits protein synthesis by acting on Elongation Factor 1A and various ribosomes. This drug’s in vitro properties generated great excitement, and several partial and complete responses were noted in Non-Hodgkin’s Lymphoma. Another Sea Squirt, *Aplidum albicans,* is the source of Aplidine, which has similar structure to Didemnin B but with less toxicity and greater antitumor activity. This drug inhibits DNA synthesis and protein synthesis. It also starves the tumor of nutrients by inhibiting secretion of VEGF, vascular endothelial growth factor. Another benefit is that cross resistance to anthracyclines, topoisomerase II inhibitors, and nucleoside analogs was not noted in cell lines of Acute Lymphoblastic Leukemia and Acute Myeloid Leukemia, demonstrating that Aplidine may have a role in both resistant forms of leukemia and leukemia’s non-responsive to other drugs [15].

The Bryozoan, *Bugula neritina*, is the source Brostatin-1 which can protect bone marrow from lethal doses of radiation, stimulate production of progenitor cells, and also stimulate the immune system to respond to malignancy. It also increases expression of p53, a molecule that encourages apoptosis of malignant cells [15]. The true origin of Bryostatin-1 underscores the complexity of ocean biodiversity and species interaction. An uncultured organism, *Candidatus endobugulasertula*, lives symbiotically with the Bryozoan and is considered to be the ultimate generator of the compound Bryostatin-1 [13].

One major component of sea organisms, polyunsaturated fatty acids, have been utilized extensively in the development of novel delivery agents for medications, serving as a key part of various solutions [11].

These chemical agents represent 8% of the total number of patents of marine genetic resources [11].

Barnacles can bind tightly to a slippery surface and hold on against the sheer force of ocean travel. Their product, “Polyphenolic protein,” allows a strong binding to metal, plastic, bone, and skin holding promise for the development of new forms of surgical adhesives [16].

Fish glide through the water with minimum friction due to either the mucous they produce or special macromolecules on their body surface. These hold promise for the medical world, where catheters, cardiac valves, and contact lenses could benefit. Of special relevance is the potential for intravascular use where these naturally derived products could prevent triggering the clotting cascade in intravascular implants [16].

The potential benefits of deep sea organisms are not confined to physical products. These organisms may provide a blueprint to better understand our biology. Researchers have been using Sea Urchin genetic code to better understand our own genome and how it relates to Alzheimer’s disease, Parkinson’s disease and various types of cancers along with several different types of muscular dystrophy [17]. Other related areas have utilized fluorescence in organisms to highlight and identify molecular processes in cells [18].

## Biology Centered Economy

With time, the efficiency of obtaining marine organisms, extracting key components and producing the molecules or raw materials will grow. Marine products will become cheaper and demand will rise. Underlying most products will be a specific novel gene discovered to code for the product of interest. Patent protection of genes will drive humanity to discover new genes and produce new marketable products. Ultimately corporate interest in new marine genes located on foreign soil or in foreign waters will raise issues of propriety and intellectual property [19]. It will open the floodgates of bioprospecting in place of biopiracy [20].

## World of Genes

In the 1950’s, the American firm Eli Lilly netted millions of dollars from the sale of two anticancer drugs obtained from Madagascar’s Rosy Periwinkle plant while the nation of origin received nothing [21]. The American firm W.R. Grace patented an insecticide from the Indian Neem tree. Riots occurred in 1993 with Indian farmers hurling accusations of “gene piracy” at the American firm [20].

International efforts to mediate such cases culminated in the 1990’s with the development of two key legislative agreements: the Trade Related Aspects of Intellectual Property Rights (TRIPS) agreement and the Biodiversity Treaty [21]. Controversy continues to surround these two pieces of legislation.

The seas are home to 34 of 36 living phyla. There are an estimated 18,000 natural products and 4,900 patents associated with genetics of marine origin and gene utilization is increasing at a rate of 12% per year. Although the number of named species from the seas increases at a rate of only 0.93% per year, the rate of new natural product discovery increases by 4%, because organisms typically each contain multiple potential products. Sponges (38%), currently contribute the most to natural products from marine resources followed by Cnidarians (20%), Tunicates (20%) and Red Algae (9%) [11]. The potential of the move from land to sea is highlighted by the 600% increase in the use of marine products from microbial organisms in 2007 compared to 1965–2005 [16]. It is estimated that one is twice as likely to find a patentable gene, and 500 times as likely to discover unknown active chemicals in marine organisms [11]. Already 55% of the genes examined have been used in the pharmaceutical and human health industry with another 26% each in, agriculture and aquaculture, 17% in food products and 7%in the cosmetics industry.

## New Underwater Techniques

Like scuba diving did for sea exploration in generations past, new technologies are making deep exploration a reality. Suits that were originally devised for space use by NASA have been adapted to for navy divers allowing for diving at high pressure, in extreme temperature zones, and areas exposed to chemical spills [22]. A new suit under investigation is using a mechanism in which a diver would breathe a highly oxygenated perflurocarbon mixture that would replace air in the lung, nose and ear cavities [23]. The lungs would extract oxygen from the mixture and carbon dioxide would be “scrubbed” from the diver by a mechanical gill embedded in the diver’s femoral vein [24]. New deep sea research habitats are allowing divers to study and take part in research for extended periods of time [24].

New vehicles have been adapted for deep exploration. The Deep sea Challenger was designed with this in mind. The vehicle includes features such as syntactic foam to withstand the high pressures, unique flotation devices, multiple forms of batteries, along with multiple backup systems that allow for safer travel [25]. In March 2013, Cameron successfully investigated Challenger Deep, the deepest area of the Mariana Trench [25]. Autonomous underwater vehicles, drones of the deep, have been developed that can be controlled by computers and artificial intelligence, [24] making deeper ocean travel more efficient.

## Economic Importance of Biotechnology

The biotechnology sector of the American economy is impressive, increasing twofold between 1993–1999, employing almost half a million people with a total impact on revenue of $46.5 billion, [26] and another three fold by 2008 with over 1.42 million employed and revenues greater than $577 billion. Further significant growth is projected by the Department of Labor and Statistics [27] Harnessing these products and the subsequent benefit to our national economy via marine biodiversity have been difficult.

Drug discoveries have been hampered by the challenge of obtaining a continued supply of a given organism as well as the culturing of such organisms. Large quantities of a compound are required for testing and development of potential drugs. Chemical synthesis is an option for the acquisition of an adequate supply of a compound once discovered.

According to some in the industry, “government must increase its involvement in marine biotechnological research” [16]. Government investment has been less in the U.S. than in other nations. China, in 2011, pledged over $308 billion to biotechnology research, while the US government struggled not to cut the $32 billion received by the NIH for biotech research. Even companies like Novartis and Pfizer are spending billions collaborating with the Chinese in huge ventures [28] that would be profiting the US if the incentives were there.

## Protecting the Future

One of the greatest threats to taking advantage of the sea’s gene potential is loss of biodiversity through our alteration and destruction of ecosystems. 48.6% of the FDA approved drugs over the past 25 years have a natural product in their history, [29] but there is concern because 30 to 50% of the world’s species face extinction by mid-century (Ibid 2004) with extinctions occurring at dozens per day [30].

A similar bounty lies within the ocean. We must not allow destruction to befall the marine world. Threats such as overfishing, dumping of nuclear and other wastes, and destruction of coastal ecosystems imperil the biodiversity of the oceans and the utilization of potential resources. Marine fisheries collapse reached 65% by 2003, and global collapse of all commercially exploited fish populations is expected by 2048 if we do not act to curb the plunder [31]. Tropical reefs shelter as many as one third of all marine species [32] but between a third and two thirds of the coral reefs are damaged or dying, causing extinction of as many as a third of reef species [33]. The coral reefs are unlikely to survive the 21^st^ century if nothing is done to prevent increasing ocean temperatures, acidification, pollution, sedimentation and direct impact from over fishing (12^th^ International Coral Reef Symposium 2012) [34]. It is vital that we act at the level of governmental and international policy to prevent irreparable damage to the last frontier even before we have the technology to benefit from the secrets of the deep.
